# PEM or MBI?

**DOI:** 10.1007/s12149-016-1077-8

**Published:** 2016-05-19

**Authors:** Paweł Zdanowski, Leszek Królicki

**Affiliations:** Nuclear Medicine Department, Public Central Teaching Hospital, Medical University of Warsaw, 1A, Banacha Str., 02-097 Warsaw, Poland

Dear Editor,

We have read with utmost interest the preliminary report by Yamamoto et al. of breast cancer screening by fluorine-18 fluorodeoxyglucose (^18^F-FDG) positron emission mammography (PEM) [1]. However, despite its many reported advantages, we have some concerns about its actual superiority in the diagnosis of tumours in the dense breast tissue. At present, there also reports on the application of another imaging modality, molecular breast imaging (MBI), for detection of breast cancer.

An increased uptake of glucose characteristic of cancer cells allows obtaining information about the metabolic activity of neoplastic tissue using ^18^F-FDG PEM. The increased uptake of FDG by cancer cells is due to the activation of GLUT 1. Subsequently the radiopharmaceutical undergoes phosphorylation and is not transported outside the cell. This phenomenon is known as the Warburg effect [2].

The degree of ^99m^Tc-MIBI uptake by the diseased tissue depends on the activity of the metabolic processes. ^99m^Tc-MIBI freely diffuses into the intracellular space and is subsequently accumulated in the mitochondria. The reverse diffusion, on the other hand, depends on the electrical potential of the mitochondrial membrane. When the metabolic activity is increased (as it is in cancer cells), the reverse diffusion is blocked. Unlike most imaging modalities, both ^18^F-FDG PEM and ^99m^Tc-MIBI do not evaluate the morphology, but provide information about the metabolic activity of neoplastic tissue.

Comparing the two modalities is of considerable clinical interest. Which of the dedicated methods is superior in the detection of breast cancer? In our analysis we took the following into consideration: imaging sensitivity and spatial resolution, dosimetric characterization, as well as the availability and cost of the examination.

The imaging sensitivity of PEM is 72–94 % with the spatial resolution of 1.0–2.4 mm. The radiation dose is 3.5 mSv. The availability is limited because PEM is not reimbursed and the cost of one examination is ca. 1100–1675 US dollars [2–4]. The imaging sensitivity of MBI is 84–96 % and the spatial resolution is 1.6–4.6 mm. The radiation dose is 2.4 mSv. The availability is better because of a lower cost of one examination (ca. 450 US dollars) [3–5].

In one-to-one comparison of the two modalities, the imaging sensitivity of MBI is higher (a difference of ca. 2 %), PEM has a better spatial resolution and detects lesions that are approximately 2.2 mm smaller, the radiation dose is lower by approximately 1.1 mSv in the case of MBI and it may be considered safer in this respect, and the cost of performing MBI is approximately 3.7 times lower than that of PEM.

Both modalities are adjuncts to mammography but the higher imaging sensitivity, lower radiation dose and lower cost per examination are the arguments in favour of MBI as a screening tool.
